# An Avascular Niche Created by Axitinib‐Loaded PCL/Collagen Nanofibrous Membrane Stabilized Subcutaneous Chondrogenesis of Mesenchymal Stromal Cells

**DOI:** 10.1002/advs.202100351

**Published:** 2021-08-28

**Authors:** Tian‐Ji Ji, Bei Feng, Jie Shen, Min Zhang, Yu‐Qing Hu, Ai‐Xia Jiang, Di‐Qi Zhu, Yi‐Wei Chen, Wei Ji, Zhen Zhang, Hao Zhang, Fen Li

**Affiliations:** ^1^ Department of Pediatric Cardiology Shanghai Children's Medical Center School of Medicine Shanghai Jiao Tong University No.1678 Dongfang Road Shanghai 200127 P. R. China; ^2^ Department of Cardiothoracic Surgery and Shanghai Institute of Pediatric Congenital Heart Disease Shanghai Children's Medical Center School of Medicine Shanghai Jiao Tong University No.1678 Dongfang Road Shanghai 200127 P. R. China; ^3^ Department of Pediatric Translational Medicine Institute Shanghai Children's Medical Center School of Medicine Shanghai Jiao Tong University No.1678 Dongfang Road Shanghai 200127 P. R. China; ^4^ Department of Cardiology The Affiliated Huaian No. 1 People's Hospital of Nanjing Medical University Jiangsu 223300 P. R. China

**Keywords:** avascular niche, axitinib, cartilage repair, electrospinning, mesenchymal stromal cells, ossification

## Abstract

Engineered cartilage derived from mesenchymal stromal cells (MSCs) always fails to maintain the cartilaginous phenotype in the subcutaneous environment due to the ossification tendency. Vascular invasion is a prerequisite for endochondral ossification during the development of long bone. As an oral antitumor medicine, Inlyta (axitinib) possesses pronounced antiangiogenic activity, owing to the inactivation of the vascular endothelial growth factor (VEGF) signaling pathway. In this study, axitinib‐loaded poly(*ε*‐caprolactone) (PCL)/collagen nanofibrous membranes are fabricated by electrospinning for the first time. Rabbit‐derived MSCs‐engineered cartilage is encapsulated in the axitinib‐loaded nanofibrous membrane and subcutaneously implanted into nude mice. The sustained and localized release of axitinib successfully inhibits vascular invasion, stabilizes cartilaginous phenotype, and helps cartilage maturation. RNA sequence further reveals that axitinib creates an avascular, hypoxic, and low immune response niche. Timp1 is remarkably upregulated in this niche, which probably plays a functional role in inhibiting the activity of matrix metalloproteinases and stabilizing the engineered cartilage. This study provides a novel strategy for stable subcutaneous chondrogenesis of mesenchymal stromal cells, which is also suitable for other medical applications, such as arthritis treatment, local treatment of tumors, and regeneration of other avascular tissues (cornea and tendon).

## Graphical abstract

Axitinib, an oral antiangiogenic medicine, is loaded into nanofibrous membranes by electrospinning. Engineered cartilage, derived from bone marrow mesenchymal stromal cells (BMSCs), is encapsulated in the membrane and subcutaneously implanted. The sustained and localized release of axitinib successfully inhibits the vascular invasion and creates an avascular niche. Consequently, stable chondrogenesis of BMSCs is achieved in the subcutaneous environment.

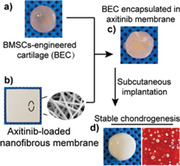

## 1 Introduction

Cartilage repair remains a problem due to the limited repair capacity of chondrocytes.^[^ ^1^ ^]^ Mesenchymal stromal cells (MSCs), which are pluripotent, and easily available, and possess self‐renewal capacity, have been proposed as a promising alternative to chondrocytes.^[^ ^1^, ^2^ ^]^ The use of MSCs has been demonstrated in articular cartilage repair;^[^ ^3^ ^]^ however, there are limitations in its use for subcutaneous cartilage formation (such as the repair of trachea cartilage): MSCs‐engineered cartilage is prone to suffer ossification and loss of the cartilaginous phenotype.^[^ ^4^, ^5^ ^]^

These limitations are possibly due to the abundant vessels existing in the subcutaneous environment while lacking blood vessels in the articular cavity. It has long been recognized that avascular cartilage invaded by blood vessels is a prerequisite for endochondral ossification during the development of long bones.^[^ ^6^, ^7^ ^]^ Moreover, articular cartilage would suffer from ossification after increased angiogenesis in arthritis.^[^ ^8^ ^]^ Typically, the process of vascular invasion during endochondral bone formation is primarily guided and facilitated by the angiogenic cues secreted by chondrocytes, such as vascular endothelial growth factor (VEGF).^[^ ^9^, ^10^, ^11^ ^]^ The inactivation of VEGF via a soluble receptor protein^[^ ^9^ ^]^ or conditional ablation of VEGF^[^ ^12^, ^13^ ^]^ in chondrocytes can successfully block vascular invasion and maintain the stability of cartilaginous phenotype, concomitant with an expanded cartilaginous zone and impaired bone formation,^[^ ^9^, ^12^, ^13^ ^]^ suggesting that VEGF plays a rather important role in endochondral ossification.

In Marsano's research, the overexpression of soluble VEGF receptors in human MSCs was shown to effectively prevent vascular invasion and promote chondrogenesis in the subcutaneous environment.^[^ ^14^ ^]^ However, apart from the carcinogenic risk carried by gene‐editing, the permanent elimination of endogenous VEGF, which plays a role in various physiological processes,^[^ ^12^ ^]^ might bring about other unexpected risks. A temporary and localized block of VEGF may be safer and more practical.

Inlyta (axitinib) is an antitumor medicine approved for the clinical treatment of advanced renal cell carcinoma.^[^ ^15^ ^]^ It is a tyrosine kinase inhibitor with highly selective inhibition of VEGF receptors.^[^ ^16^ ^]^ Owing to the inactivation of the VEGF signaling pathway, axitinib can significantly inhibit the proliferation, migration, and tube formation of endothelial cells and present excellent antiangiogenic activity for the treatment of malignant tumors.^[^ ^17^, ^18^ ^]^ Furthermore, compared to the monoclonal antibody against VEGF (bevacizumab),^[^ ^19^ ^]^ axitinib is a novel, oral, small‐molecule, antiangiogenic drug (C22H18N4OS, Mw: 386.47) that is not only more cost‐effective but also has considerably higher chemical stability.

Electrospinning is widely used in drug delivery.^[^ ^20^ ^]^ High surface area, good permeability, and adjustable degradation rate give electrospun nanofibers an excellent ability for the localized and sustained release of various drugs.^[^ ^21^ ^]^ Poly(*ε*‐caprolactone) (PCL), a synthetic polymer with excellent mechanical properties, has been wildly used as a carrier in drug delivery systems;^[^ ^22^ ^]^ however, its application is restricted by its slow degradation rate, poor cell adhesion, and proliferation rate.^[^ ^21^ ^]^ On the other hand, collagen is a natural polymer with good biocompatibility but poor mechanical properties and a rapid degradation rate.^[^ ^23^ ^]^ Therefore, the electrospun nanofibrous membrane composed of synthetic polymer PCL and native polymer collagen might be an ideal vehicle for the delivery of axitinib.

**Figure 1:**
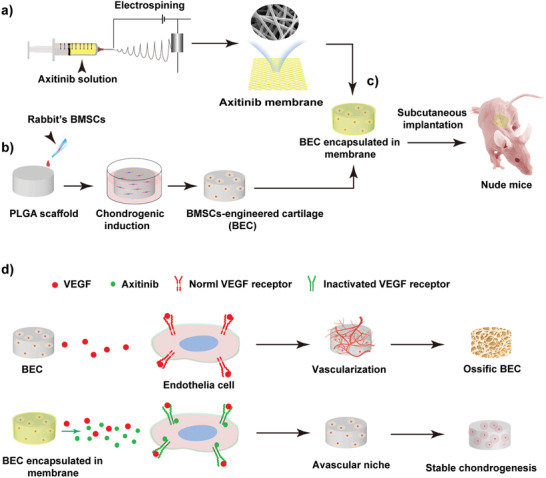
Schematic illustration of the experimental design. a) Axitinib‐loaded PCL/collagen nanofibrous membranes were fabricated by electrospinning. b) The construction of BEC. c) The encapsulation and subcutaneous implantation of BEC. d) Sustained and localized release of axitinib could effectively inhibit vascularization, creating an avascular niche for stable chondrogenesis of BEC.

In this study, MSCs‐engineered cartilage was encapsulated in the axitinib‐loaded PCL/collagen electrospun membrane and subcutaneously implanted, as shown in **Figure** **1**. The sustained and localized release of axitinib successfully prevented the vascular invasion and created an avascular, hypoxia, and low immune response niche. As a result, stable chondrogenesis of MSCs was achieved in the subcutaneous environment.

## 2 Results and Discussion

### 2.1 Schematic Illustration of the Experimental Design

The axitinib‐loaded PCL/collagen nanofibrous membrane was prepared by electrospinning (Figure 1a). Bone marrow MSCs (BMSCs) isolated from the femur of New Zealand white rabbits^[^ ^24^ ^]^ were seeded into poly (lactic‐*co*‐glycolic acid) (PLGA) scaffolds and then cultured in the chondrogenic medium^[^ ^5^ ^]^ for 4 weeks to prepare BMSCs‐engineered cartilage (BEC) (Figure 1b). Then, BEC was encapsulated in the axitinib‐loaded nanofibrous membrane and subcutaneously implanted into nude mice (Figure 1c). The sustained and localized release of axitinib could effectively inhibit the vascularization, creating an avascular niche for stable chondrogenesis of BEC (Figure 1d).

### 2.2 Characterizations of Axitinib‐Loaded PCL/Collagen Membranes

**Figure 2:**
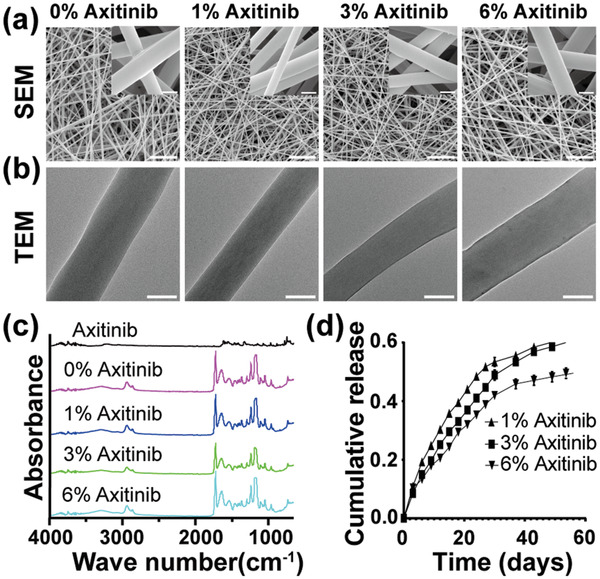
Characterizations of axitinib‐loaded PCL/collagen nanofibrous membranes. a) Representative SEM images of different nanofibrous membranes. Scale bars were 10 µm (low magnification) and 500 nm (high magnification). b) Representative TEM images of different nanofibrous membranes. Scale bar = 500 nm. c) FTIR spectra of axitinib and different electrospun membranes. d) Axitinib release behavior in different electrospun membranes.

The morphology of different membranes was observed using a scanning electron microscope (SEM). The incorporation of axitinib had no significant influence on the fiber morphology, with smooth and uniform nano‐microstructures in all groups (**Figure** **2**a). Transmission electron microscope (TEM) images showed that axitinib was evenly distributed in the nanofibrous membranes without drug nanoparticle agglomeration (Figure 2b). This might because axitinib is a small, fat‐soluble molecule that could be quickly dissolved in HFIP to form a transparent solution (Figure S1, Supporting Information). The nanofiber diameter in each group was similar (700.1 ± 120.1, 636.1 ± 124.5, 646.3 ± 135.4, and 723.8 ± 124.4 nm for 0%, 1%, 3%, and 6%‐Axitinib groups, respectively) (Figure S2a, Supporting Information). All membranes showed a hydrophilic surface with similar contact angles at the beginning, which decreased quickly and almost completely disappeared within 5 s (Figure S2b, Supporting Information). According to the Fourier transform infrared (FTIR) spectra of different membranes, the characteristic peaks of bovine tendon type I collagen appeared at ≈1660 cm^−1^ (amide I) and 1550 cm^−1^ (amide II). The PCL‐related characteristic peaks were evident at 2943 cm^−1^ (asymmetric CH2 stretching), 2866 cm^−1^ (symmetric CH2 stretching), and 1721 cm^−1^ (C═O stretching). The encapsulation of axitinib had no significant influence on the chemical structure of the PCL/collagen membranes (Figure 2c).

The encapsulation efficiency was 89.42 ± 0.77% (1%‐Axitinib), 85.15 ± 1.72% (3%‐Axitinib), and 80.67 ± 0.65% (6%‐Axitinib), and showed a decreasing trend with increasing axitinib content. The release behaviors of axitinib in the different membranes were generally stable and slow; the more the axitinib encapsulated in the membranes, the slower the release profiles observed. The cumulative release rate of axitinib was up to 61.30 ± 3.17% (1%‐Axitinib), 60.79 ± 2.81% (3%‐Axitinib), and 49.59 ± 1.44% (6%‐Axitinib) on day 56 (Figure 2d).

### 2.3 The Bioactivity and Biocompatibility of Axitinib‐Loaded PCL/Collagen Membranes In Vitro

**Figure 3:**
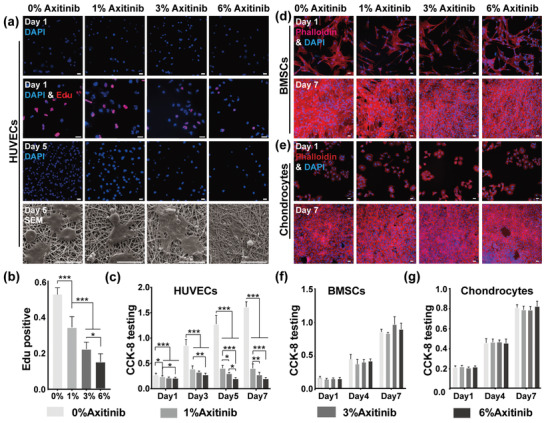
Bioactivity and biocompatibility of axitinib‐loaded PCL/collagen membranes. a) The bioactivity of axitinib‐loaded PCL/collagen membranes nanofibrous on HUVECs. Nuclei staining and Edu staining (Edu‐594, in red) were performed on day 1. Nuclei staining and SEM were performed on day 5. Blue: nucleus. Red: proliferating nucleus. Scale bar = 20 µm. b) The statistical analysis of Edu positive percentage of HUVECs seeded on different membranes on day 1. Values represent mean ± SD, *n* = 6, *P < 0.05, ***P < 0.001. c) CCK‐8 testing of HUVECs cultured on axitinib‐loaded membranes at different time points. Values represent mean ± SD, n = 5, *P < 0.05, **P < 0.01, ***P < 0.001. The biocompatibility of axitinib‐loaded PCL/collagen nanofibrous membranes with BMSCs d) and chondrocytes e). Nuclei and cytoskeleton staining of BMSCs and chondrocytes cultured on different membranes were performed on day 1 and day 7. Blue: nucleus. Red: cytoskeleton. Scale bar = 20 µm. CCK‐8 testing of BMSCs f), and chondrocytes g) cultured on axitinib‐loaded membranes at different time points. Values represent mean ± SD, *n* = 5.

To evaluate the bioactivity of axitinib encapsulated in membranes, human umbilical vein endothelial cells (HUVECs) were seeded onto different membranes.^[^ ^18^ ^]^ After 1 day of incubation, the cell numbers adhering to different membranes were similar, whereas cell proliferation was quite different, as shown by the Edu assay (**Figure** **3**a). 52.95 ± 3.76% of HUVECs were proliferating in the 0%‐Axitinib group, while only 34.44 ± 5.99% in the 1%‐Axitinib group and ≈20% in the 3%‐Axitinib and 6%‐Axitinib groups (Figure 3b). As a result, obvious growth inhibition was observed on membranes containing axitinib 5 days after cell seeding (Figure 3a). Specifically, HUVECs in the 0%‐Axitinib and 1%‐Axitinib groups were round and smooth with a 3D structure, while the shape of HUVECs became flat and fragmented in the 3%‐Axitinib and 6%‐Axitinib groups, as observed by SEM (Figure 3a). When we further analyzed the proliferation of HUVECs by CCK‐8 testing, the inhibition with the increasing content of axitinib was more obvious (Figure 3c).

Since axitinib was used to stabilize the chondrogenesis of BMSCs‐engineered cartilage in the subcutaneous environment, the biocompatibility of axitinib‐loaded membranes with BMSCs and chondrocytes were evaluated. Before seeding, BMSCs at passage 2 were identified by flow cytometry. More than 98% of cells were positive for CD44 and CD90, whereas almost no CD11b‐ and CD45‐positive positive cells were identified (Figure S3, Supporting Information), which agreed with previous reports.^[^ ^25^ ^]^

BMSCs and auricular chondrocytes (isolated from rabbits) adhered and spread well on different membranes without obvious differences on day 1. As the incubation time increased, BMSCs and chondrocytes exhibited robust proliferation and finally reached ≈100% confluency on day 7, with bright fluorescence (Figure 3d,e). Further evaluation of cell activity by CCK‐8 testing revealed no significant differences in the absorbance among different groups (Figure 3f,g), indicating that despite the strong inhibition of endothelial cells, axitinib‐loaded membranes showed good biocompatibility with BMSCs and chondrocytes.

### 2.4 The Construction, Encapsulation, and Implantation of BEC

**Figure 4:**
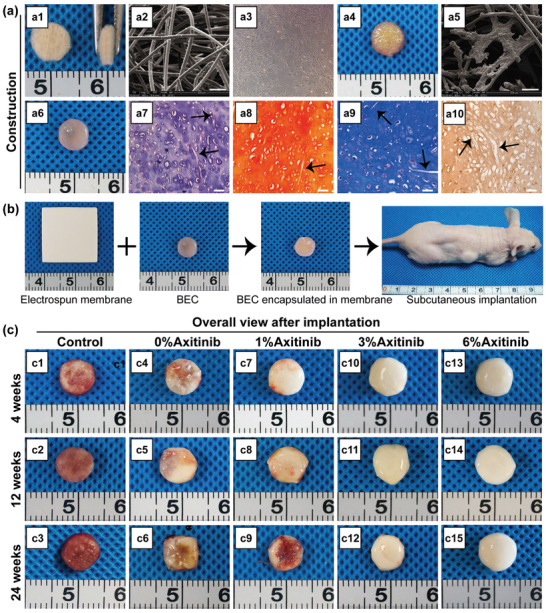
Construction and subcutaneous implantation of BEC. a) Construction and evaluation of BEC in vitro. a1) Overall view of the PLGA scaffold. a2) Representative SEM image of the PLGA scaffold. a3) Morphology of BMSCs at passage 2. a4) Overall view after BMSCs were seeded into the PLGA scaffold. a5) SEM image one day after the seeding of BMSCs. a6) Overall view of BEC after 4 week chondrogenic induction. a7–a10) Representative images of pathological staining of BEC sections, including hematoxylin and eosin (HE) staining a7), safranin O/fast green staining a8), Masson's trichrome staining a9), and immunohistochemistry staining of collagen II a10). Scale bar = 50 µm. Black arrows represented residual PLGA fibers. b) The encapsulation of BEC in axitinib‐loaded nanofibrous membranes and subcutaneous implantation into nude mice. c) Overall view of BEC from each group after 4, 12, and 24 weeks of implantation. Control group: BEC alone c1–c3); 0%‐Axitinib group c4–c6); 1%‐Axitinib group c7–c9); 3%‐Axitinib group c10–c12); 6%‐Axitinib group c13–c15).

To construct BEC, unwoven PLGA fibers were chosen as scaffolds, which were porous and shapable with a highly uniform fiber diameter (**Figure** **4**a1,4a2). BMSCs at passage 2 were seeded into PLGA scaffolds and adhered well on day 1 (Figure 4a3–4a5). After 4‐week chondrogenic induction, BEC formed and presented a smooth hyaline, and cartilage‐like appearance (Figure 4a6). According to the pathological analysis, BEC presented typical cartilage features with abundant lacuna‐like structures and positive and homogenous staining for safranin O, Masson's trichrome staining, and collagen II, along with abundant residual PLGA fibers (Figure 4a7–4a10). Then BEC was encapsulated in axitinib‐loaded membranes and subcutaneously implanted into nude mice (Figure 4b; and Figure S4, Supporting Information). BEC alone was implanted into nude mice as a control group.

After 4, 12, and 24 weeks of implantation, BEC was retrieved from all the groups. As shown in Figure 4c1–4c3, samples in the control group showed obvious vascular invasion even at the early stage of implantation and finally presented a reddish‐brown appearance after 24 week implantation. Compared with the control group, the vascular invasion was partly prevented in the 0%‐Axitinib and 1%‐Axitinib groups (Figure 4c4–4c9). Interestingly, samples in the 3%‐Axitinib and 6%‐Axitinib groups presented a smooth and ivory‐whitish appearance throughout the implantation (Figure 4c10–4c15), indicating that axitinib at a concentration of 3% could sustainably resist subcutaneous vascular invasion.

### 2.5 Stable Chondrogenesis of Engineered Cartilage in the Avascular Niche Created by Axitinib

**Figure 5:**
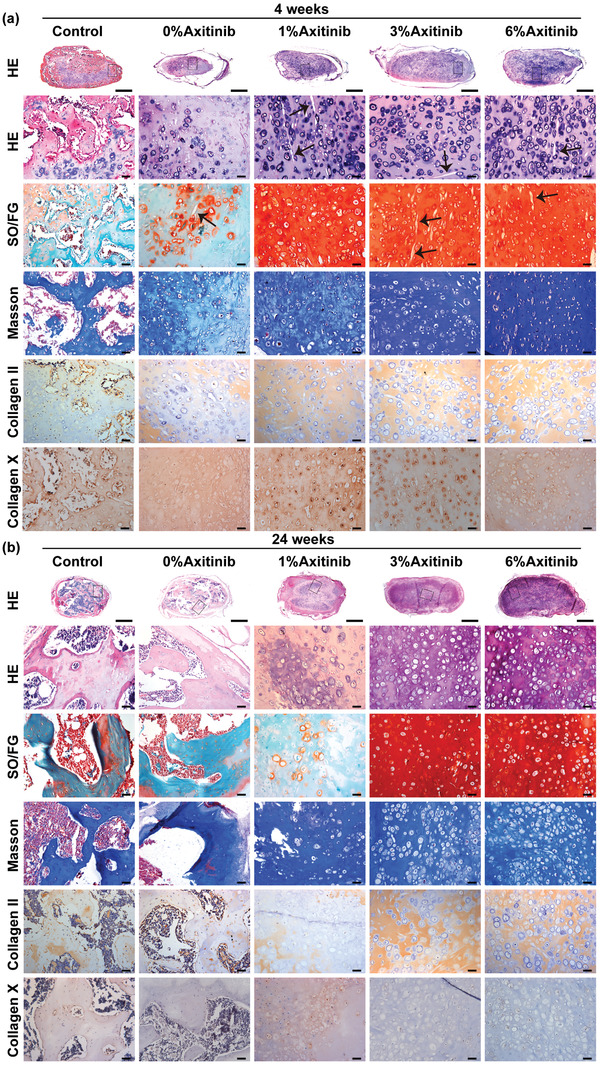
Histological and immunohistochemistry staining of BEC in different groups at 4 a) and 24 weeks b) postimplantation. Tissue sections were stained with hematoxylin and eosin (HE), safranin O/fast green (SO/FG), and Masson's trichrome reagents. Chondrogenic marker‐collagen II and hypertrophic marker‐collagen X were evaluated by immunohistochemistry staining. Scale bars: 1 mm (low magnification) and 50 µm (high magnification). Black arrows represented residual PLGA fibers.

According to the pathological analysis, after 4 weeks of subcutaneous implantation, samples in the control group mainly consisted of bone‐like tissues with positive fast green staining, and cartilage tissue only existed in some central areas where positive safranin O and collagen II staining was observed. No typical bony structures were observed in the 0%‐Axitinib group, revealing that membranes, as a physical barrier, played a part role in maintaining the cartilaginous phenotype. However, samples in the axitinib groups (1%, 3%, and 6%) maintained stable cartilage repair, which presented abundant lacuna‐like structures with positive and homogeneous staining of cartilage‐specific extracellular matrix components (safranin O and collagen II) and weak positive staining of hypertrophic hallmark (collagen X) (**Figure** **5**a). After a 12 week prolonged subcutaneous implantation, samples in the 0%‐Axitinib group began to form typical bone‐like tissues with partial fibrosis, similar to those observed in the control group, indicating that BEC was prone to lose its phenotype after subcutaneous implantation. Samples in the 1%‐Axitinib group also gradually lost their cartilage features, replaced with ossified or fibrous tissues, concomitant with the weakened safranin O and collagen II staining and inhomogeneous Masson's trichrome staining. Meanwhile, the PLGA fibers also gradually degraded with a prolonged implantation time and were invisible after 12 weeks of subcutaneous implantation (Figure S5, Supporting Information).

Surprisingly, the samples maintained their cartilage features even after 24 weeks of subcutaneous implantation in the 3%‐Axitinib and 6%‐Axitinib groups. Typical mature hyaline chondrocytes and cartilage lacunas were observed all over the samples at low magnification. Strong positive and homogeneous staining of safranin O and collagen II were observed, while nearly negative staining of collagen X, revealing stable chondrogenesis in the avascular niche. The general statistics of the in vivo fate of BEC in each group are shown in Table S1, Supporting Information.

**Figure 6:**
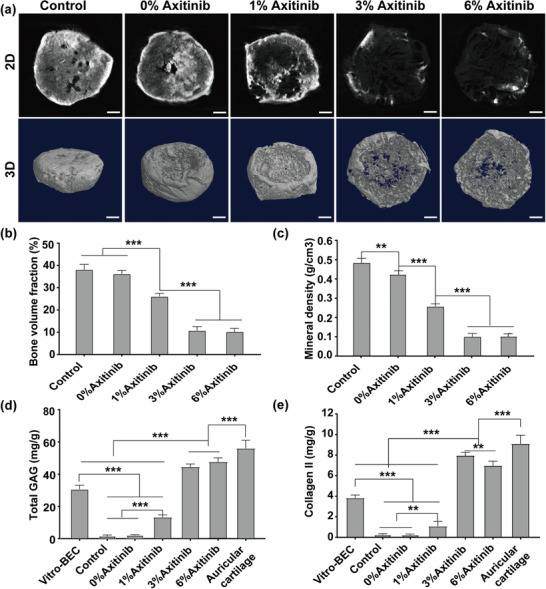
Micro‐CT evaluation and chondrogenesis assessment of BEC at 24 weeks postimplantation. a) Representative 2D and 3D images of BEC by micro‐CT in different groups. Scale bar = 1 mm. The bone volume fraction b) and mineral density c) of BEC. Values represented mean ± SD, *n* = 3, **P < 0.01, ***P < 0.001. Quantification of cartilage‐specific extracellular matrix GAG d) and Collagen II e) in different groups. BEC before implantation was used as a basic control, and native auricular cartilage was used as a positive control. Values represented mean ± SD, *n* = 3, **P < 0.01, ***P < 0.001.

Stable chondrogenesis was further confirmed by micro‐CT.^[^ ^26^, ^27^ ^]^ After 24 weeks of implantation, significant calcification was detected in whole samples in the control and 0%‐Axitinib groups, while calcified areas were mainly found in the peripheral regions in the 1%‐Axitinib group. However, scarce calcification was detected at the edge of samples in the 3%‐Axitinib and 6%‐Axitinib groups (**Figure** **6**a). Further, quantitative analysis showed that bone volume fraction and bone mineral density in the 3%‐Axitinib and 6%‐Axitinib groups were significantly lower than those in the other groups (Figure 6b,c).

The quantitative analysis of glycosaminoglycan (GAG) and collagen II was also encouraging. Compared with those in BEC before implantation, the contents of GAG and collagen II decreased in the 1%‐Axitinib group and were almost undetectable in the control group and 0%‐Axitinib group. However, the contents were significantly higher in the 3%‐Axitinib and 6%‐Axitinib groups, nearly reaching the levels in native auricular cartilage (Figure 6d,e), indicating that stable subcutaneous cartilage tissues could be obtained when the content of axitinib reached 3%.

### 2.6 Evaluation of Self‐Stability of Engineered Cartilage

Axitinib was an exogenous antivascular substance that was released completely over time. Whether the engineered cartilage remained stable over time or underwent ossification is an important issue.

**Figure 7:**
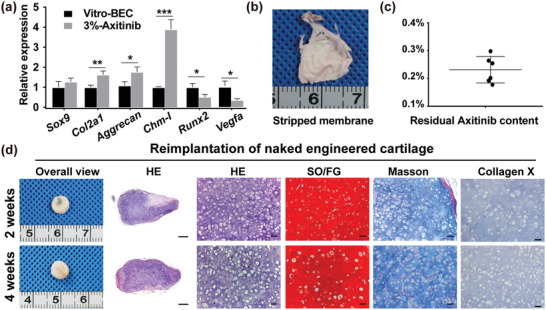
Evaluation of self‐stability of engineered cartilage. a) Relative mRNA expression levels of chondrogenesis (*Sox9, Col2a1, Aggrecan*), proangiogenesis (*Runx2, Vegfa*), and antiangiogenesis (*Chm‐I*) were comparatively analyzed between vitro‐BEC and BEC in the 3%‐Axitinib group (in vivo 24 weeks). Values represent mean ± SD, *n* = 3, *P < 0.05, **P < 0.01, ***P < 0.001. b) After 20 weeks of implantation, the membrane surrounding BEC in the 3%‐Axitinib group was stripped. c) Residual axitinib in the stripped membrane was measured by high‐performance liquid chromatography. Values represent mean ± SD, *n* = 6. d) Naked engineered cartilage, derived from 3%‐Axitinib group (in vivo 20 weeks), was reimplanted subcutaneously into another nude mouse and harvested at 2 or 4 weeks postimplantation. Tissue sections were stained with hematoxylin and eosin (HE), safranin O/fast green (SO/FG), and Masson's trichrome reagents. Hypertrophic marker‐collagen X were evaluated by immunohistochemistry staining. Scale bars: 1 mm (low magnification) and 50 µm (high magnification).

Mature chondrocytes can secrete various antiangiogenic cues, such as Chm‐I and endostatin, to resist vascular invasion and maintain a stable cartilaginous phenotype.^[^ ^28^ ^]^ Once *Chm‐I* is knocked out, native cartilage also suffers vascularization and ossification after subcutaneous implantation.^[^ ^26^ ^]^ Based on the qPCR analysis between vitro‐BEC and BEC in the 3%‐Axitinib group (in vivo 24 weeks), apart from genes related to chondrogenesis (*Col2a1* and *Aggrecan*), the expression of antiangiogenic gene (*Chm‐I*) was remarkably upregulated in the 3%‐Axitinib group. In contrast, the expression of endogenous angiogenic gene (*Vegfa*), expressed by chondrocytes,^[^ ^9^, ^10^, ^12^, ^13^ ^]^ as well as *Runx2* (the upstream regulator of *Vegfa*), was downregulated (**Figure** **7**a), which was further confirmed by immunohistochemistry of Vegfa (Figure S6, Supporting Information). These results indicated that the inhibition of vascular invasion might not only aid the stabilization of phenotype but also help engineered cartilage maturation.

Further, to judge whether the engineered cartilage was able to remain stable alone, we stripped the membrane surrounding the engineered cartilage in the 3%‐Axitinib group (in vivo 20 weeks). Residual axitinib in the membrane was measured by high‐performance liquid chromatography and naked engineered cartilage was implanted subcutaneously into a new nude mouse. As shown in Figure 7b,c, at 20 weeks after implantation in vivo, the membrane remained obvious and the residual axitinib content was around 0.23% ± 0.04% ($\frac{\mathrm{weight}\;\mathrm{of}\;\mathrm{residual}\;\mathrm{axitinib}\;}{\;\mathrm{total}\;\mathrm{weight}\;\mathrm{of}\;\mathrm{residual}\;\mathrm{membrane}}$). This meant that only 7.7% of axitinib was unreleased compared with the initial payload. Besides, the naked engineered cartilage resisted vascularization and maintained a stable cartilaginous phenotype without a hypertrophic phenotype, even at an extra 2 or 4 weeks after implantation (Figure 7d). Thus, at 20 weeks after implantation, the engineered cartilage in the 3% axitinib group had become sufficiently mature to remain stable itself for at least 4 weeks, even without the aid of axitinib. These results demonstrated that our strategy was effective for obtaining stable subcutaneous cartilage tissues. Whether the naked engineered cartilage remains stable over a longer period will be investigated in our next study.

### 2.7 The Avascular, Hypoxia, and Low Immune Response Niche Created by Axitinib, Upregulated the Expression of Timp1 and Inhibited the Matrix Metalloproteinases (MMPs) Activity

**Figure 8:**
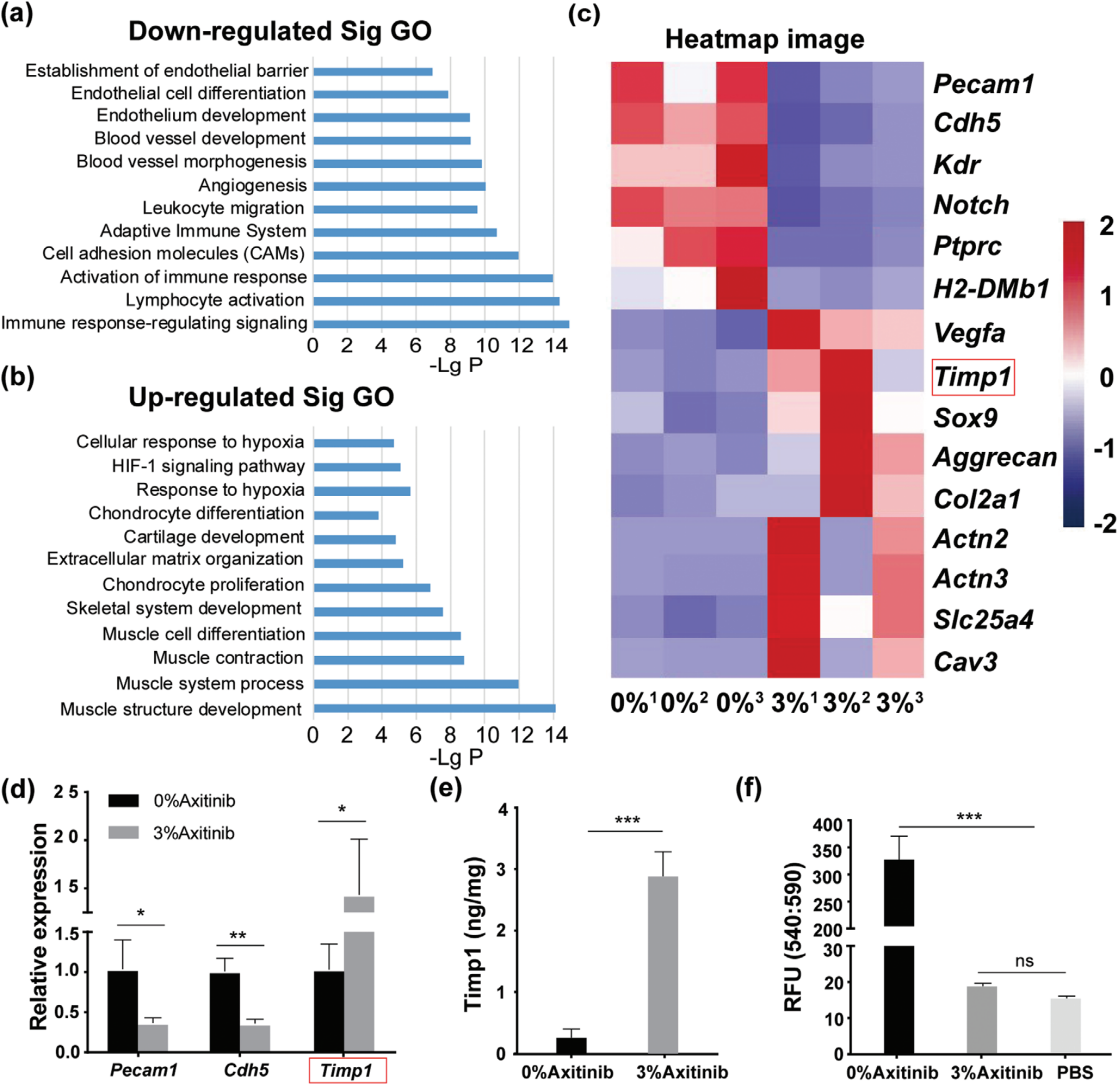
Transcriptional analysis of murine tissues surrounding BEC and protein validation tests in the 0%‐Axitinib and 3%‐Axitinib groups at 12 weeks postimplantation. Go term analysis of down‐regulated a) and up‐regulated b) gene‐related enrichment signaling pathway. c) Heatmap image of related gene expression. The scale is −2 to +2 on the logarithmic scale. d) Relative mRNA expression of angiogenic genes (*Pecam1*, *Cdh5*), and *Timp1* (an endogenous inhibitor of MMPs) by qPCR. Values represent mean ± SD, *n* = 3. *P < 0.05, **P < 0.01. e) The quantification of murine Timp1 by Elisa. Values represent mean ± SD, *n* = 5, ***P < 0.001. f) The MMP activity assay. The relative fluorescence units (RFU) were monitored using a microplate reader with a filter set of Ex/Em = 540/590 nm. Values represent mean ± SD, *n* = 5. ***P < 0.001, ns indicated no significance.

To better understand how axitinib affects the maturation of BEC through the niche, transcriptional profiles of murine tissues surrounding BEC in the 0%‐Axitinib group and 3%‐Axitinib group were comparatively analyzed by high‐throughput sequencing. Among the 20 318 genes analyzed, 837 genes were differentially expressed by more than 1.5‐fold, with 313 genes downregulated and 524 genes upregulated in the 3%‐Axitinib group. Gene ontology (GO) analysis revealed that the genes downregulated in the 3%‐Axitinib group were mainly enriched in endothelial development, angiogenesis, and immune response (**Figure** **8**a). Genes upregulated in the 3%‐Axitinib group were mainly enriched in response to hypoxia, Hif‐1 signaling pathway, cartilage differentiation and development, and muscle structure development (Figure 8b).

The heatmap and qPCR analysis further showed that the expression of angiogenic genes decreased significantly, including *Pecam1*, *Cdh5*, *Kdr*, and *Notch4* (Figure 8c,d), and this result was further confirmed by the immunohistochemistry of Pecam1 (CD31) (Figure S7, Supporting Information). Surface marker of leukocytes (*Ptprc*) and various histocompatibility antigens (such as *H2‐DMb1*) were also downregulated (Figure 8c), which was further confirmed by the immunohistochemistry of Ptprc (CD45) (Figure S8, Supporting Information). These results reflected a weakened immune response. In contrast, the expression of genes related to hypoxia (such as *Vegfa* and *Timp1*) and chondrogenic markers (*Sox9*, *Aggrecan*, and *Col2a1*) was significantly upregulated (Figure 8c). As shown in Figure 8c, genes (Actn2, Actn3, Slc25a4, Cav3) related to muscle contraction and ATP synthesis,^[^ ^29^ ^]^ were significantly upregulated in the 3%‐Axitinib group. Considering muscle tissue is sensitive to the oxygen content in the microenvironment,^[^ ^30^ ^]^ this increased transcriptional activity was likely an adaptive response of muscle tissue surrounding the engineered cartilage, to the hypoxia niche created by axitinib.

Normally, the invaded vasculature recruits a series of cells in charge of cartilage absorption and bone deposition, such as osteoclasts and osteoblasts.^[^ ^7^, ^10^, ^31^ ^]^ These cells secrete various MMPs (osteoclasts: Mmp9, osteoblasts: Mmp13) to accomplish the matrix remodeling.^[^ ^31^ ^]^ Mice deficient in *Mmp9* and *Mmp13* present a dramatically expanded chondrocyte zone and severely impaired bone formation,^[^ ^32^ ^]^ demonstrating the important role of these MMPs in endochondral ossification. However, despite the successful inhibition of vascularization, neither Mmp9 nor Mmp13 showed significant downregulation in the 3%‐Axitinib group (Figures S9 and S10, Supporting Information). Apart from the enzyme concentration, the MMP activity is also regulated by tissue inhibitors of metalloproteinases (TIMPs).^[^ ^33^ ^]^ In this study, as a response to hypoxia,^[^ ^34^ ^]^ *Timp1* was remarkably upregulated in the 3%‐Axitinib group (Figure 8c,d). Furthermore, the protein validation tests confirmed the upregulation of Timp1 (Figure 8e; and Figure S10, Supporting Information) and suppressed MMP activity (Figure 8f) in the 3%‐Axitinib group, suggesting a possible correlation between Timp1 and MMP activity.

To elucidate the relationship between Timp1 and MMP activity, a series of in vivo experiments were performed. Electrospun membranes were tailored into rectangles (15 × 15 mm^2^), disinfected, and subcutaneously implanted into nude mice. After 2 weeks of implantation, samples were isolated and evaluated by the overall view, Timp1 content, and MMP activity.

As shown in Figure S11 (Supporting Information), murine tissues surrounding the 3%‐Axitinib membrane presented inhibited vascularization and MMP activity but upregulated Timp1 as compared with those in the 0%‐Axitinib group. The extra addition of Timp1 could effectively suppress the vascularization and MMP activity in the 0%‐Axitinib group, mimicking the phenotype in the 3%‐Axitinib group. Moreover, when Timp1 was functionally blocked by a neutralizing antibody, angiogenesis was partly improved in the 3%‐Axitinib group, concomitant with elevated MMP activity. Based on these results, we concluded that Timp1 possibly played a functional role in the action of axitinib by inhibiting the MMP activity.

Usually, cells and drugs are encapsulated in the same biomaterial (such as hydrogels),^[^ ^35^ ^]^ which is simple and convenient. In this study, unlike TGF*β* and Kartogenin,^[^ ^36^ ^]^ which are directly involved in the chondrogenic differentiation of MSCs, axitinib indirectly promotes chondrogenesis of MSCs by inhibiting peripheral vascular invasion. Considering VEGF also matters for the survival of chondrocyte itself,^[^ ^12^ ^]^ the release of axitinib from the edge of constructs can minimize its adverse effects on seed cells, which can better determine the relationship between antiangiogenesis and in vivo stable chondrogenesis. On the other hand, although it can be stripped off as need, the presence of a membrane surrounding the engineered cartilage might hamper the integration of the engineered tissue into the surrounding native tissue. This situation can be improved by adjusting the degradation rate of the membranes. Either changing the ratio of collagen in the PCL/collagen membrane or replacing PCL with easily degradable materials (such as PLGA and PLCL) will be feasible.

## 3 Conclusion

In summary, axitinib‐loaded biodegradable PCL/collagen nanofibrous membranes were prepared and used for subcutaneous cartilage formation in this study. The nanofibrous membrane successfully mimics the protective effect of the articular cavity on chondrogenesis, blocking peripheral vascular invasion without impeding the transport of nutrients. With the temporary help of this membrane, MSCs engineered cartilage manages to get maturation and finally gets rid of the dependence on exogenous antiangiogenic drugs. Moreover, for the first time, the functionality of TIMP1 is proven to be one plausible mechanism for stable chondrogenesis within the avascular niche in vivo, providing new ideas or new targets for further study. This study provided a novel strategy for stable subcutaneous cartilage formation of mesenchymal stromal cells (such as the repair of trachea cartilage), which could also be used in other medical applications such as arthritis treatment, local treatment of tumors, and regeneration of other avascular tissues (cornea and tendon).

## 4 Experimental Section

Details about materials and experimental methods are available in Supporting Information. GEO: GSE160825.

## Conflict of Interest

The authors declare no conflict of interest.

## Supporting information

Supporting information

## Data Availability

The data that support the findings of this study are openly available in Gene Expression Omnibus database at https://www.ncbi.nlm.nih.gov/geo/query/acc.cgi?acc=GSE160825, reference number GSE160825.
