# Correction to “An Avascular Niche Created by Axitinib‐Loaded PCL/Collagen Nanofibrous Membrane Stabilized Subcutaneous Chondrogenesis of Mesenchymal Stromal Cells”

**DOI:** 10.1002/advs.202511638

**Published:** 2025-08-14

Tianji Ji, Bei Feng, Jie Shen, Min Zhang, Yuqing Hu, Aixia Jiang, Diqi Zhu, Yiwei Chen, Wei Ji, Zhen Zhang, Hao Zhang, Fen Li. An Avascular Niche Created by Axitinib‐Loaded PCL/Collagen Nanofibrous Membrane Stabilized Subcutaneous Chondrogenesis of Mesenchymal Stromal Cells. Adv Sci (Weinh). 2021 Oct;8(20): e2100351.

https://doi.org/10.1002/advs.202100351

Fluorescent images of the 6%‐Axitinib group on day 7 in Figure 3d, 6%‐Axitinib group on day 1 in Figure 3e, and 3%‐Axitinib group on day 7 in Figure 3e were misused due to a large volume of raw data; we have replaced them with the correct ones.


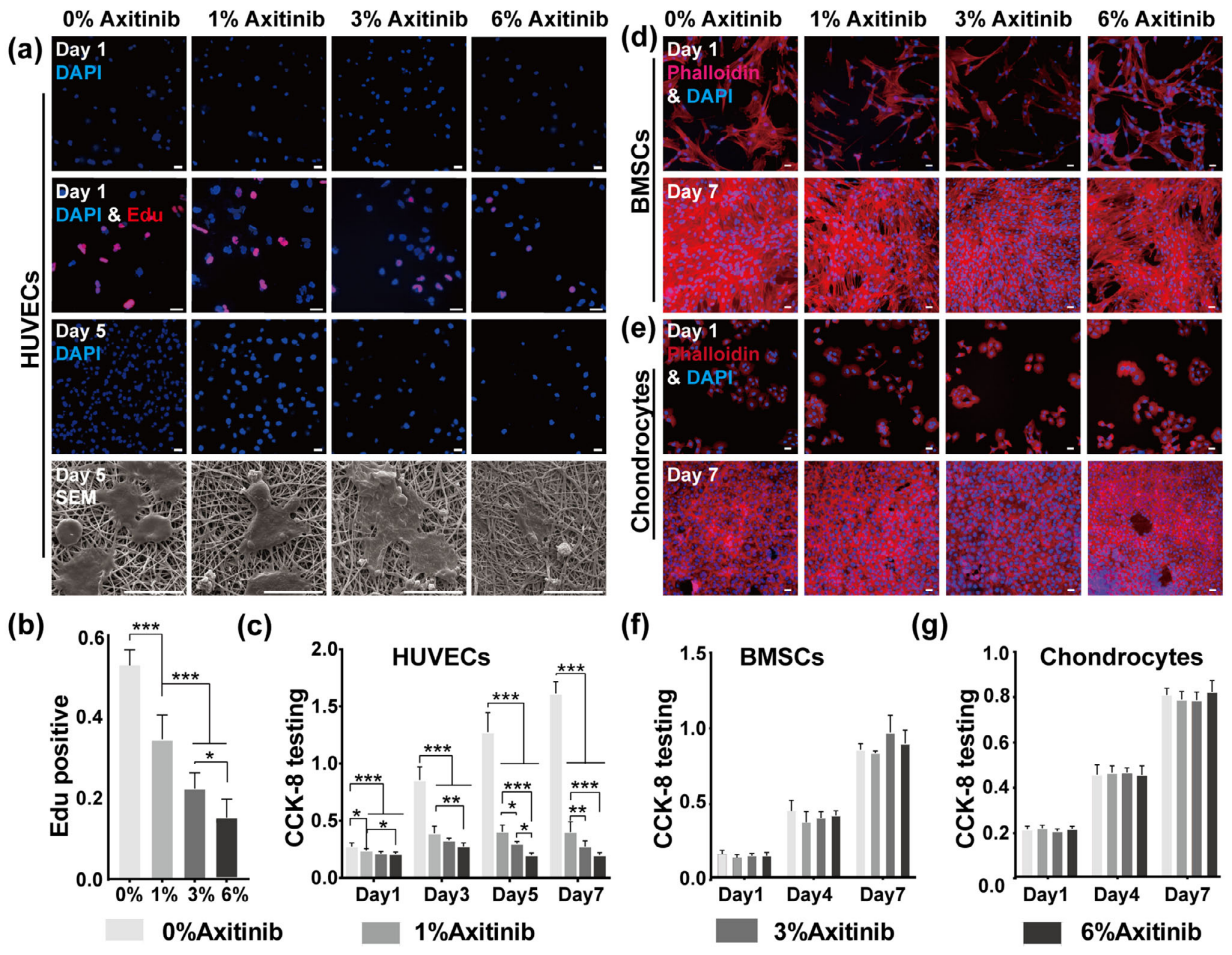


We have thoroughly rechecked all data in the main document and Supporting Information, confirming that the corrections do not affect the description or scientific conclusions of the article.

We apologize for this error.

## Supporting information



Supporting Information

