# Supplementary material for: An Avascular Niche Created by Axitinib‐Loaded PCL/Collagen Nanofibrous Membrane Stabilized Subcutaneous Chondrogenesis of Mesenchymal Stromal Cells
Source: Adv Sci (Weinh). 2021 Aug 28;8(20):2100351. doi: 10.1002/advs.202100351 (PMC8529489; doi:10.1002/advs.202100351)
Supplement: Supplementary file 1 — Supporting information [file ADVS-8-2100351-s001.pdf]

© 2021 Wiley-VCH GmbH

ADVANCED SCIENCE Open Access

Supporting Information

for *Adv. Sci.,* DOI: 10.1002/advs.202100351

An Avascular Niche Created by Axitinib-Loaded PCL/Collagen Nanofibrous Membrane Stabilized Subcutaneous Chondrogenesis of Mesenchymal Stromal Cells

Tian-Ji Ji, Bei Feng, Jie Shen, Min Zhang, Yu-Qing Hu, Ai-Xia Jiang, Di-Qi Zhu, Yi-Wei Chen, Wei Ji, Zhen Zhang, Hao Zhang,* and Fen Li**

An avascular niche created by axitinib-loaded PCL/collagen nanofibrous membrane stabilized subcutaneous chondrogenesis of mesenchymal stromal cells

Tian-Ji Ji[ξ], *Bei Feng*[ξ]*, *Jie Shen*[ξ], *Min Zhang, Yu-Qing Hu, Ai-Xia Jiang, Di-Qi Zhu, Yi-Wei Chen, Wei Ji, Zhen Zhang, Hao Zhang*, Fen Li**

E-mail: Bei Feng (fengbei@scmc.com.cn), Hao Zhang (zhang-hao@scmc.com.cn), Fen Li (*lifen@scmc.com.cn*)

ξ These authors contributed equally to this work.

Experimental section

Animals: Male New Zealand white rabbits (2-months-old, n=10) were purchased from the Songlian Experimental Animal Raising Farm (Shanghai, China). Male nude mice (6–8 weeks old, n=68) were purchased from the Lingchang Biotechnology Company (Shanghai, China). All experimental procedures were approved by the Animal Care and Experiment Committee of the Shanghai Jiao Tong University School of Medicine.

Materials: PCL (average Mw: 80,000; 440744), collagen (from bovine Achilles tendon; C9879), collagenase (C6885), dexamethasone (D4902), and ascorbic acid (A4544) were purchased from Sigma Aldrich (USA). 1,1,1,3,3,3-Hexafluoro-2-propanol (HFIP, 165540) was purchased from J&K Chemicals (China). Axitinib (M1658) was purchased from AbMole Bioscience (USA). α-MEM (22571038), DMEM with high glucose (11965092), antibiotic-antimycotic (100X, 15240062), fetal bovine serum (FBS; 10099141C), trypsin

(15050057), and phosphate buffer saline (PBS; 10010049) were purchased from Thermo Scientific (USA). Serum substitute (HPCPLCRL50) was purchased from Helios Bioscience (USA). Cell Counting Kit-8 (CCK-8; CK04) was purchased from Dojindo Laboratories (Japan). The Edu Cell Proliferation Kit (C0078) was purchased from Beyotime Biotechnology (China). Non-woven fabric poly (lactic-co-glycolic acid) (PLGA-1090) scaffolds were

purchased from Equl Ltd (USA), which were composed of PLA and PGA at a mass ratio of 10:90. Recombinant human TGFβ1 (240-B) and IGF1 (291-G1) were purchased from R&D system (USA). Insulin-Transferrin-Selenium (ITS) (100X, ITSS-10201) was purchased from Cyagen Biosciences (USA). Antibodies for flow cytometry: CD11b (MCA802GA), CD44 (MCA806GA), CD45 (MCA808GA) and CD90 (MCA47A488T) were purchased from Bio-rad Laboratories (USA).

Fabrication of axitinib-loaded PCL/collagen nanofibrous membranes: PCL and collagen were dissolved in HFIP at a mass ratio of 60/40 and a concentration of 12% (wt/v), and then stirred at room temperature for 24 h. Then, 1 h before electrospinning, axitinib was added to the solution at mass ratios of 0%, 1%, 3%, and 6% ($\frac{\text{weight of axitinib}}{\text{total weight of PCL and collagen}}$). The electrospinning conditions were as follows: injection rate was 1.5 mL/h, 30% to 50% wet, the voltage was 10 kV, and distance between the syringe needle and the grounded rotating plate (300 rpm) was 11 cm. Nanofibrous membranes were vacuum freeze-dried for 48 h and then preserved at -40 °C before use.

Characterization of PCL/collagen nanofibrous membranes: The surface morphologies of different membranes were observed using a scanning electron microscope (SEM; TM 3030 Plus, Hitachi). More than 100 random nanofibers were measured by Nano Measurer software to determine the average fiber diameter. The nanofibrous structures and distribution of axitinib were observed using a transmission electron microscopy (TEM; JEM2100, Japan).

The hydrophilicity of the different membranes was evaluated using a hydrophilic angle tester (Attension Theta, Biolin Scientific AB, Sweden). Deionized water (5 µL) was dropped

carefully onto the surface of different membranes. The contact angle was measured at random locations.

Attenuated total reflection Fourier transform infrared spectroscopy (ATR-FTIR) of axitinib powder and nanofibrous membranes were performed using a Nicolet-670 FTIR spectrometer (Thermo Scientific, USA). All spectra were recorded at the 1000–4000 cm^{-1} wavelength range.

The encapsulation efficiency of axitinib was measured as follows:[1] a known mass of the membrane was dissolved in 1 mL HFIP. 100 uL of the solution was then added to 2 mL of methanol and centrifuged to remove the precipitated PCL and collagen. 20 uL of the diluted solution was detected using a high-performance liquid chromatography (HPLC) instrument (1260 Infinity II Prime, Agilent, USA) at λ = 330 nm. The amount of axitinib was obtained from the standard curve of axitinib. The encapsulation efficiency was calculated using the following equation:

$$\text{Encapsulation efficiency}=\frac{\text{weight of axitinib in the sample}}{\text{theoretical weight of axitinib loading in the sample}}\ 100\%$$

The release profile of axitinib was determined by soaking the membranes in PBS in vitro. The membranes were cut into squares, accurately weighed, and incubated in 5 mL PBS at 37 °C while shaking mildly. Every 3 to 6 days, all solutions were collected for HPLC detection to determine the exact amount of axitinib released, which was then replaced with an extra 5 mL PBS. The cumulative release rate was calculated based on the actual weight of the axitinib encapsulated in the membranes.

Cell isolation and culture: Human umbilical vein endothelial cells (HUVECs) were

purchased from Cyagen Biosciences (USA). The culture medium for HUVECs was composed of DMEM with high glucose, 10% FBS, and antibiotic-antimycotic (1X).

BMSCs and chondrocytes were isolated from New Zealand white rabbits.[2] Briefly, harvested bone marrow was washed, centrifuged, and resuspended in the α-MEM enrichment medium containing 5% serum substitute, 1 unit/mL heparin, and antibiotic-antimycotic (1X). The medium was replaced every 2 to 3 days until the primary BMSCs reached a confluence of approximately 80%. Then, the BMSCs were collected by 0.25% trypsin and subcultured at a density of 1×10^4 cells/cm^2. BMSCs at passage 2 were used for further experiments.

BMSCs at passage 2, were identified by fluorescence-activated cell sorting (FACS). Briefly, cells were resuspended at a density of 1×10^6 cells/mL in PBS. Then four different anti-rabbit antibodies (CD11b, CD44, CD45, CD90) were added into the suspensions respectively. Finally, the positive fractions of different cell surface markers were analyzed by a flow cytometry (BD, USA)

Auricular cartilage tissues were digested with 0.25% trypsin at 37 °C for 30 min, cut into 1 mm^3 pieces, and then digested with 0.1% collagenase at 37 °C for 1.5 h. Primary chondrocytes were collected, resuspended, and seeded onto dishes at a density of 2×10^4 cells/cm^2 in the same medium used for the HUVECs. Chondrocytes at passage 2 were used for further experiments.

Bioactivity of axitinib released from PCL/collagen membranes: Membranes were tailored into round shapes (15 mm in diameter) and sterilized under UV irradiation for 30 min. Subsequently, membranes were placed at the bottom of 24-well culture plates and fixed with

steel rings. The HUVECs were seeded on the membranes at a density of 1 × 10^4 cells/well.

1 or 5 days after cell seeding, HUVECs were washed thrice with PBS, fixed with 2.5% glutaraldehyde, and stained with DAPI. The cell density of HUVECs was directly observed using a fluorescent inverted microscope (DMI300B, Leica, Germany).

The Edu assay was performed 1 day after cell seeding to evaluate the cell proliferation potential. Briefly, 30 μM Edu was added to the cell culture medium and incubated with HUVECs for 2 h at 37 °C. HUVECs were fixed, permeabilized, and stained with Azide-594 and then counterstained with DAPI. The percentage of Edu-positive cells was analyzed using a fluorescent inverted microscope. More than 6 high-power fields were countered to determine the proliferation potential of HUVECs on different membranes.

$$\text{Edu positive} = \frac{\text{cells stained with red}}{\text{all stained cells}} \text{ X } 100\%$$

The morphology of HUVECs was also observed via SEM. Briefly, 5 days after seeding, HUVECs seeded on membranes were fixed with 2.5% glutaraldehyde, dehydrated with graded alcohols, and dried naturally. Then, samples were coated with gold and observed under an electron microscope.

To further evaluate the proliferation of HUVECs, CCK-8 testing was performed 1, 3, 5, and 7 days after cell seeding. Briefly, HUVECs were incubated with CCK-8 kit (Dojindo, Japan) for 2 h at 37 °C. Then, absorbance at 450 nm was measured using a microplate reader (Synergy2, Bio Tek, USA).

Biocompatibility of axitinib-loaded PCL/collagen membranes with BMSCs and chondrocytes: Membranes were prepared as described above. BMSCs or chondrocytes were

then seeded onto membranes at a density of 1×10^4 or 2×10^4 cells/well, respectively.

To directly analyze the morphological features of BMSCs or chondrocytes on membranes, the cytoskeleton was stained with phalloidin (PHDH1, Cytoskeleton, USA) and counterstained with DAPI 1 day or 7 days after cell seeding. To evaluate the proliferation activity, the CCK-8 test was performed 1, 4, and 7 days after cell seeding.

Construction and evaluation of the vitro-BEC: The PLGA scaffolds were tailored and compressed into cylindrical shapes (6 mm diameter and 2 mm depth) and then disinfected with 75% ethanol solution for 1 h and washed thrice with PBS. Thereafter, 2nd passage BMSCs were suspended in enrichment medium, as previously described,[3] to a final concentration of 6×10^7 cells/mL, and 65 µL of the cell suspension was evenly seeded into each scaffold, followed by a 5 h incubation. After two days of cultivation in the enrichment medium, the constructs were cultured in the chondrogenic medium composed of DMEM with high glucose, 10 ng/mL TGFβ1, 100 ng/mL IGF1, ITS (1X), 25 µg/mL ascorbic acid, 40 ng/mL dexamethasone, and antibiotic-antimycotic (1X) for 4 weeks to prepare the BEC. Finally, the chondrogenesis in the BEC was evaluated through histological examination.

Encapsulation of the vitro-BEC and implantation in vivo: The BEC was encapsulated in PCL/collagen nanofibrous membranes with a small amount of tissue adhesive (Histoacryl, B. Braun Melsungen AG, Germany). First, BEC was placed onto the center of the membrane. Second, the four corners of the membrane were folded to the upper surface of the BEC. Finally, 4–8 µL Histoacryl was dripped on the folded corners.

The constructs containing the BEC were divided into five groups and implanted

subcutaneously into nude mice: 1) Control group: BEC alone; 2) 0%-Axitinib group: BEC encapsulated in membranes; 3) 1%-Axitinib group: BEC encapsulated in membranes containing 1% axitinib; 4) 3%-Axitinib group: BEC encapsulated in membranes containing 3% axitinib; 5) 6%-Axitinib group: BEC encapsulated in membranes containing 6% axitinib. Two constructs of the same group were subcutaneously implanted into the dorsal side of one mouse. After 4, 12, and 24 weeks of implantation, 6 samples from each group at one time were harvested for histological analysis.

Histology and immunohistochemistry: Samples were fixed in 10% formalin, decalcified in 10% EDTA, embedded in paraffin, and sectioned. To evaluate the histological structure and cartilage-specific extracellular matrix deposition, sections were stained with hematoxylin and eosin (HE), safranin O/fast green (SO/FG), and Masson's trichrome. The expression of collagen II was evaluated using a mouse anti-rabbit monoclonal antibody (NB600-844, Novus, USA). The expression of Collagen X was evaluated using a rabbit anti-rabbit polyclonal antibody (bs-0554R, Bioss, China). The expression of Vegfa was detected using a mouse anti-rabbit monoclonal antibody (ab1316, Abcam, USA). The expression of Pecam1 (CD31) was examined using a rat anti-mouse monoclonal antibody (NB600-1475, Novus, USA) to evaluate vascular invasion. The expression of Ptprc (CD45) was detected using a rat anti-mouse monoclonal antibody (NB100-77417, Novus, USA) to evaluate leukocyte infiltration. The distribution of Mmp9, Mmp13, and Timp1 were evaluated respectively by different rabbit anti-mouse antibodies (bs-4593R/bs-0575R/bs-0415R, Bioss, China).

Micro-CT: After 24 weeks of subcutaneous implantation, samples (n=3 for each group) were

fixed in 10% formalin and stored in 75% ethanol at 4 °C before measurement.[4] The micro-CT (SkyScan 1076, Belgium) settings were as follows: pixel matrix, 1024 × 1024; slice thickness: 8 μm; resolution: 8 μm. After scanning, a three-dimensional histo-morphometric analysis was performed automatically. Total mineral density (g/cm^3) and bone volume fraction were measured for quantitative evaluation.

Chondrogenesis assessment of the engineered cartilage: The glycosaminoglycan (GAG) and Collagen II contents were chosen as the indices of chondrogenesis. After 24 weeks of subcutaneous implantation, samples were harvested from each group. The GAG content was quantified using dimethyl methylene blue chloride (DMMB, Sigma, USA),[2] and the Collagen II content was quantified using an ELISA kit (ml1001622-C, Enzyme-linked Biotechnology, Shanghai, China), according to the manufacturer's instructions. Vitro-BEC was used as a basal control, while native auricular cartilage was considered as a positive control.

Evaluation of self-stability of engineered cartilage: mRNA expression levels of engineered cartilage were analyzed in the 3%-Axitinib group (*in vivo* 24 weeks, n=3) and compared with those of vitro-BEC (n=3). Rabbit *Sox9*, *Col2a1*, *Aggrecan*, *Chm-I*, *Runx2*, and *Vegfa* were chosen to estimate the chondrogenesis and ossification tendency, with *beta-actin* (*Actb*) was used as an internal control. Primer sequences were shown in Table S2, supporting information.

Further, to judge whether the engineered cartilage was able to remain stable alone, we stripped the membrane surrounding the engineered cartilage in the 3%-Axitinib group (in vivo

20 weeks, n=6).The residual axitinib in the membrane was measured by HPLC and the nude engineered cartilage was subcutaneously implanted into a new nude mouse. After 2 or 4 weeks of implantation, samples were harvested and evaluated by the overall view and pathological staining.

RNA sequence and qPCR analysis of murine tissues surrounding engineered cartilage: After 12 weeks of subcutaneous implantation, samples from the 0%-Axitinib group and 3%-Axitinib group were harvested, with the murine tissues entangled. Total RNA was extracted using Trizol and evaluated by agarose gel electrophoresis and Agilent 2100 Bioanalyzer (USA). After the RNA samples were qualified, mRNAs were enriched using magnetic beads with Oligo (dT). Isolated mRNAs were then fragmented into short pieces, reverse transcribed into cDNA, purified, terminally repaired, ligated to the adapter, and amplified. Raw mRNA sequence data were obtained using an Illumina HiSeq™2000 machine (USA) and filtered using Fastp software.

First, the trimmed reads were mapped to the mouse genome (mm10, genome.UCSC.edu) using the STAR software to identify the transcriptional information in the niche of BEC. Then, the DESeq2 “counts” function of the R package was used to normalize gene expression levels based on the number of total reads of each sample. Differentially expressed genes between the 0%-Axitinib group and 3%-Axitinib group were identified as meeting the criteria of fold change ≥ 1.5 and P-value < 0.05. Lastly, we used Metascape (http://metascape.org) for gene ontology analysis.

For qPCR analysis, murine mRNAs were reverse transcribed into cDNA. Primers for

target genes (*Pecam1*, *Cdh5*, *Timp1*, *Mmp9* and *Mmp13*) were designed, shown in Table S3, supporting information. PowerUp™ SYBR™ Green Master Mix (Thermo Scientific, USA) was used to amplify the specific cDNAs. The results were analyzed using the comparative threshold cycle (△△CT) method with *Actb* as an internal control.

Quantification of Timp1 and measurement of MMP activity: The whole samples were immediately flash-frozen in liquid nitrogen, homogenized by a ceramic mortar, and dissolved in PBS at a concentration of 5 mg/mL of tissues. The samples were centrifuged at 12000 rpm for 10 min at 4 °C and the supernatant was collected. The content of Timp1 was determined using an ELISA kit (MTM100, RD, USA) according to the recommendations of the manufacturer. The general MMP activity of the samples was measured using a fluorometric MMP activity assay kit (ab112147, Abcam, USA).[5] First, 2 mM 4-aminophenylmercuric acetate (APMA) was added to an equal volume of supernatant and incubated at 37 °C for 3 h to activate MMPs. Next, the MMP red substrate working solution was added to the activated MMP solution. Finally, the fluorescence intensity was monitored using a fluorescence plate reader (Synergy2, Bio Tek, USA) at Ex/Em= 540/590 nm after incubation at 37 °C for 60 min.

In vivo modulation of Timp1: To elucidate the relationship between Timp1 and MMP activity, a series of in-vivo experiments were performed. Electrospun membranes were tailored into rectangles (15 mm * 15 mm), disinfected, and subcutaneously implanted into nude mice. Samples were divided into five groups according to the axitinib content and modulation of Timp1: 1) 0%-Axitinib group: 0%-Axitinib membrane alone; 2) 0%-Axitinib +

Timp1: Nude mice loaded with 0%-Axitinib membrane received hypodermic injections of recombinant mouse Timp1 (35 μg/kg) (980-MT, R&D, USA); 3) 3%-Axitinib group: 3%-Axitinib membrane alone; 4) 3%-Axitinib + Timp1 block: Nude mice loaded with 3%-Axitinib membrane received hypodermic injections of a function-blocking antibody of murine Timp1 (0.5 mg/kg) (AF980, R&D, USA); 5) 3%-Axitinib + Ig G: Nude mice loaded with 3%-Axitinib membrane received hypodermic injections of an isotype IgG (0.5 mg/kg) (AB-108-C, R&D, USA). Four pieces of the electrospun membranes were subcutaneously implanted into two nude mice in each group. The injections started immediately after the implantation and were repeated every two days for two weeks. All related reagents were injected at locations surrounding the membrane.

Statistical analysis: All quantitative data were obtained from no less than three samples and presented in terms of their mean ± standard deviation. The Anderson–Darling test was first performed to evaluate if the raw data fit the normal distribution curve. If this fit did not exist, logarithmic transformation was done on this data. A Student's t-test was used to determine statistical differences between two groups, while a one-way analysis of variance (ANOVA), followed by a Tukey's test, was conducted for multiple group comparisons. Significant differences were assumed at p-values below 0.05 (*p < 0.05; **p < 0.01; ***p < 0.001). All statistical analyses were performed using the software SPSS Statistics 24.0 (IBM, USA).

Reference

[1] J. Xue, M. He, H. Liu, Y. Niu, A. Crawford, P. D. Coates, D. Chen, R. Shi, L. Zhang, *Biomaterials* **2014**, *35*, 9395.

[2] X. He, B. Feng, C. Huang, H. Wang, Y. Ge, R. Hu, M. Yin, Z. Xu, W. Wang, W. Fu, J. Zheng, *International journal of nanomedicine* **2015**, *10*, 2089.

[3] D. Li, L. Zhu, Y. Liu, Z. Yin, Y. Liu, F. Liu, A. He, S. Feng, Y. Zhang, Z. Zhang, *Acta biomaterialia* **2017**, *54*, 321.

[4] Y. Zhu, Y. Zhang, Y. Liu, R. Tao, H. Xia, R. Zheng, Y. Shi, S. Tang, W. Zhang, W. Liu, Y. Cao, G. Zhou, *Tissue engineering. Part A* **2015**, *21*, 782.

[5] C. Boada, A. Zinger, C. J. Tsao, P. Zhao, J. O. Martinez, K. Hartman, T. Naoi, R. Sukhovershin, M. Sushnitha, R. Molinaro, *Circ. res* **2019**.

Supplementary Figures

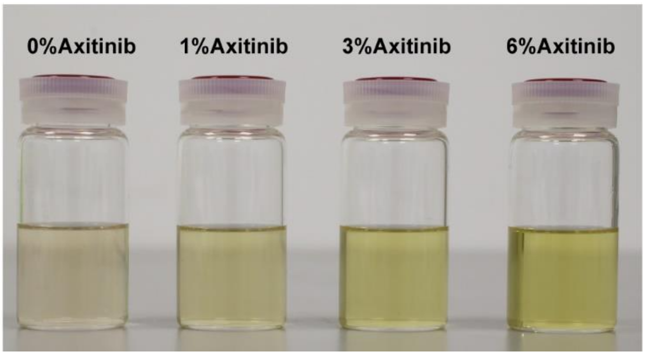

Figure S1. Samples containing different content of axitinib dissolved in 12% (wt/v) PCL/collagen/HFIP electrospinning solution.

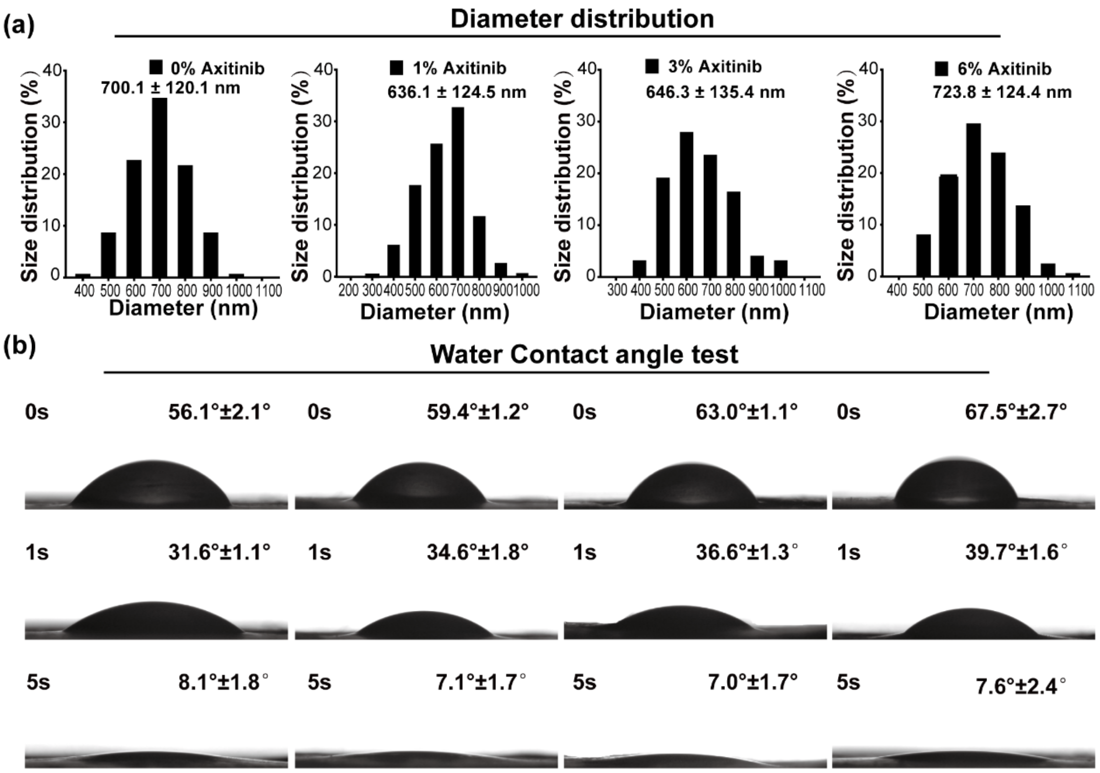

Figure S2. Characterizations of axitinib-loaded PCL/collagen membranes. (a) Diametric distribution of different nanofibrous membranes. Values represent the mean ± SD, n = 100. (b) Representative images of the water contact angle. Values represent the mean ± SD, n = 6.

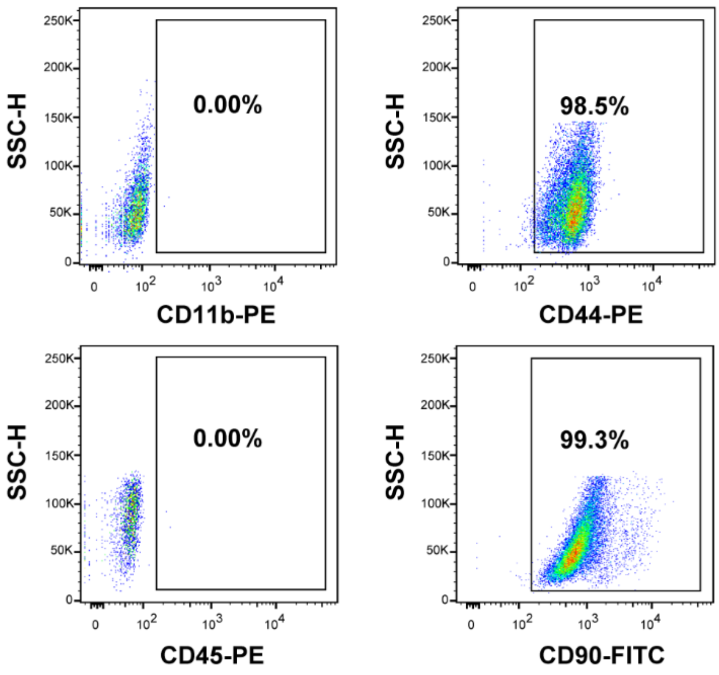

Figure S3. Identification of BMSCs by flow cytometry. More than 98% of cells were positive for CD44 and CD90, whereas almost no CD11b- and CD45-positive cells were identified, indicating the high purity of mesenchymal stromal cells.

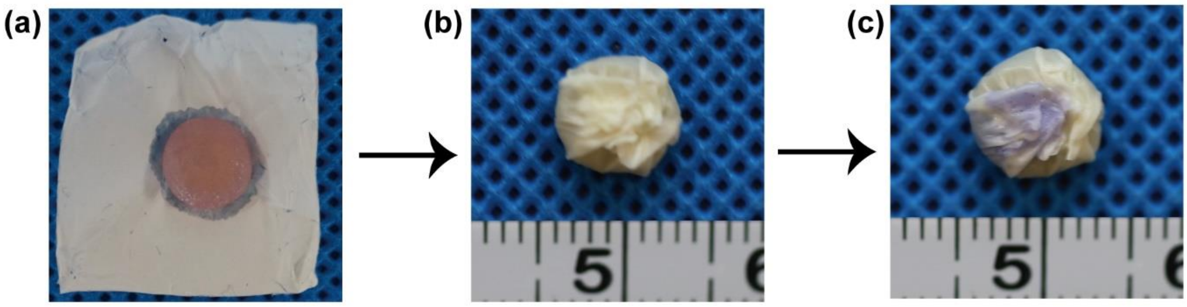

Figure S4. Schematic diagrams of the encapsulation of BEC. (a) BEC was placed onto the center of the membrane. (b) The four corners of the membrane were folded to the upper surface of the BEC. (c) 4–8 µL Histoacryl was dripped on the folded corners. Blue areas in the membrane represented the Histoacryl.

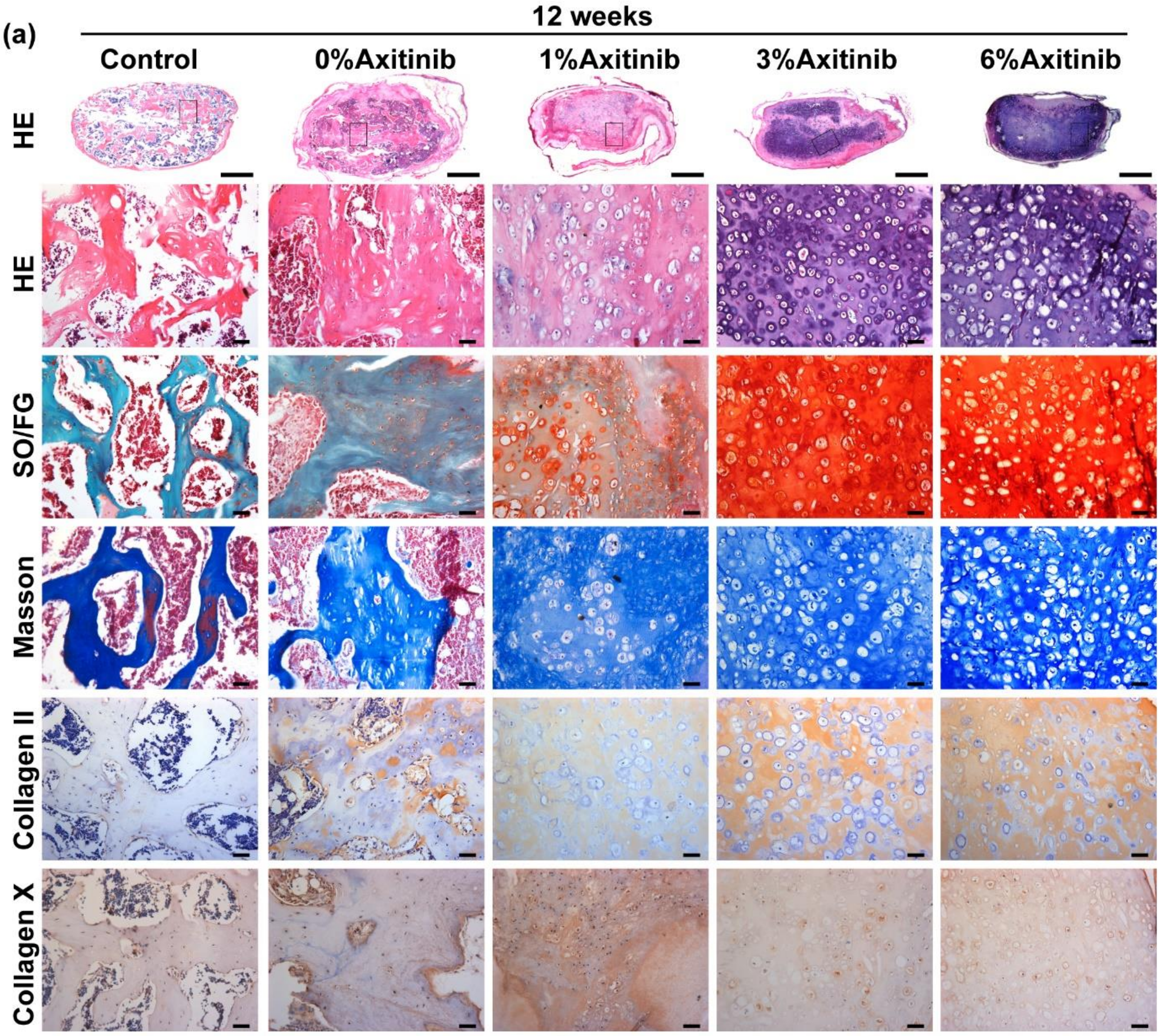

Figure S5. Histological and immunohistochemistry staining of BEC in different groups at 12 weeks post-implantation. Tissue sections were stained with hematoxylin and eosin (HE), safranin O/fast green (SO/FG), and Masson's trichrome reagents. Chondrogenic marker-collagen II and hypertrophic marker-collagen X were evaluated by

immunohistochemistry staining. Scale bars: 1 mm (low magnification) and 50 μm (high magnification).

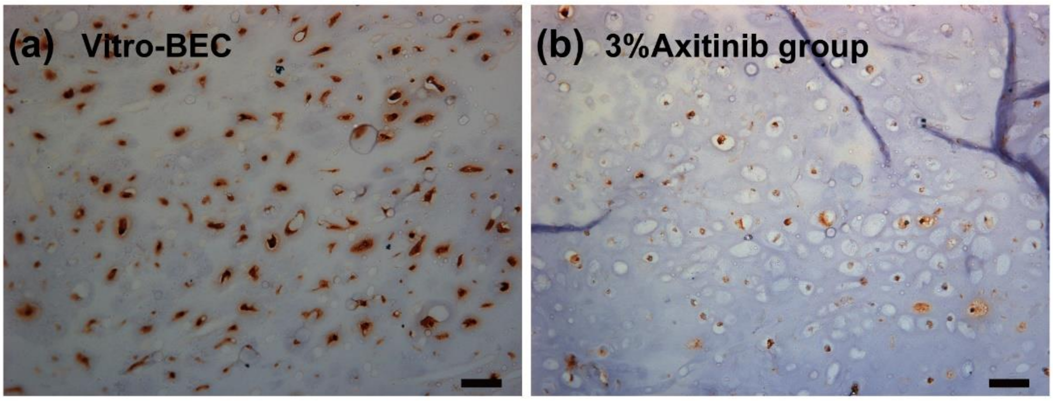

Figure S6. Immunohistochemistry staining of Vegfa in vitro-BEC and 3%-Axitinib group (*in vivo* 24 weeks). (a) Significantly positive expression of Vegfa detected in BEC before implantation. (b) Remarkably weakened expression of Vegfa in 3%-Axitinib group after subcutaneous implantation. Scale bar = 50 μm.

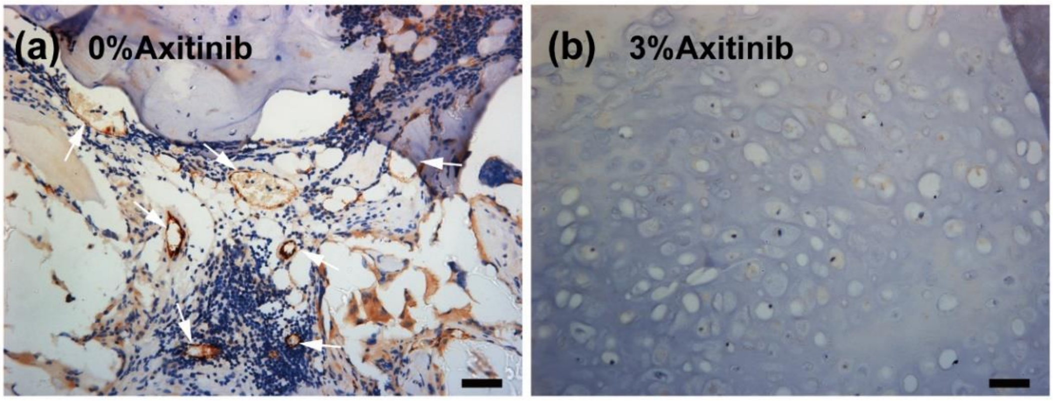

Figure S7. Immunohistochemistry staining of Pecam1 (CD31) in 0%-Axitinib and 3%-Axitinib groups at 12 weeks post-implantation. (a) Vascular invasion was observed in the 0%-Axitinib group. White arrows represented blood vessels. (b) The absence of vessels was observed in the 3%-Axitinib group. Scale bar = 50 μm.

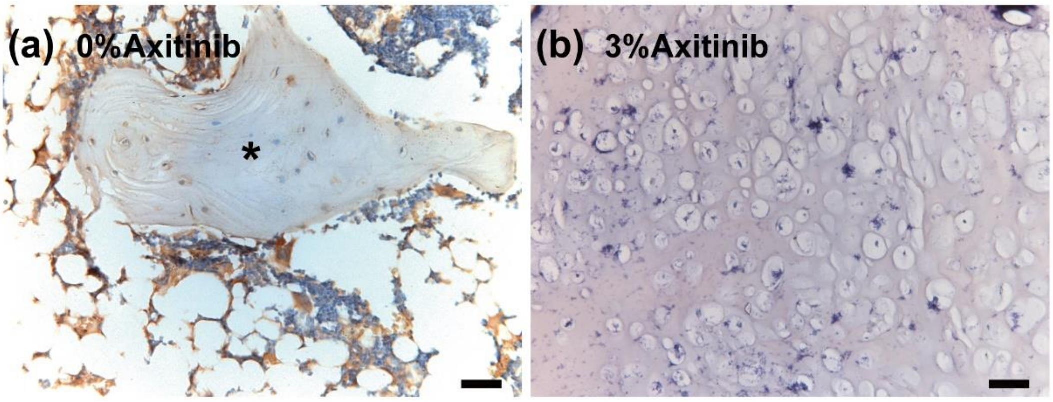

Figure S8. Immunohistochemistry staining of Ptprc (CD45) in 0%-Axitinib and 3%-Axitinib groups at 12 weeks post-implantation. (a) Abundance leukocytes infiltrating the engineered cartilage was observed in the 0%-Axitinib group. * represented ossific engineered cartilage. (b) No obvious leukocytes were observed in the 3%-Axitinib group. Scale bar = 50 μm.

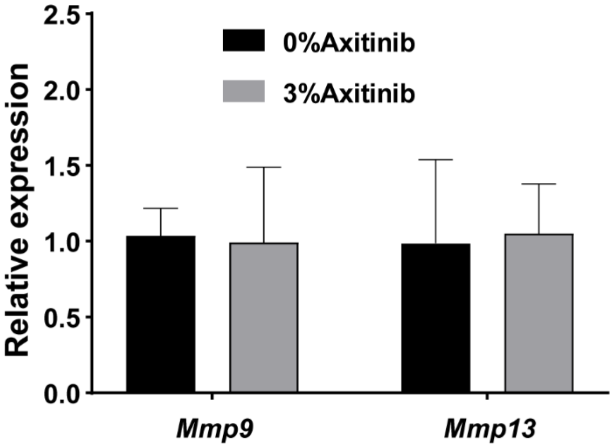

Figure S9. Relative mRNA levels of murine *Mmp9* and *Mmp13* in 0%-Axitinib and 3%-Axitinib groups at 12 weeks post-implantation. No significant difference was observed.

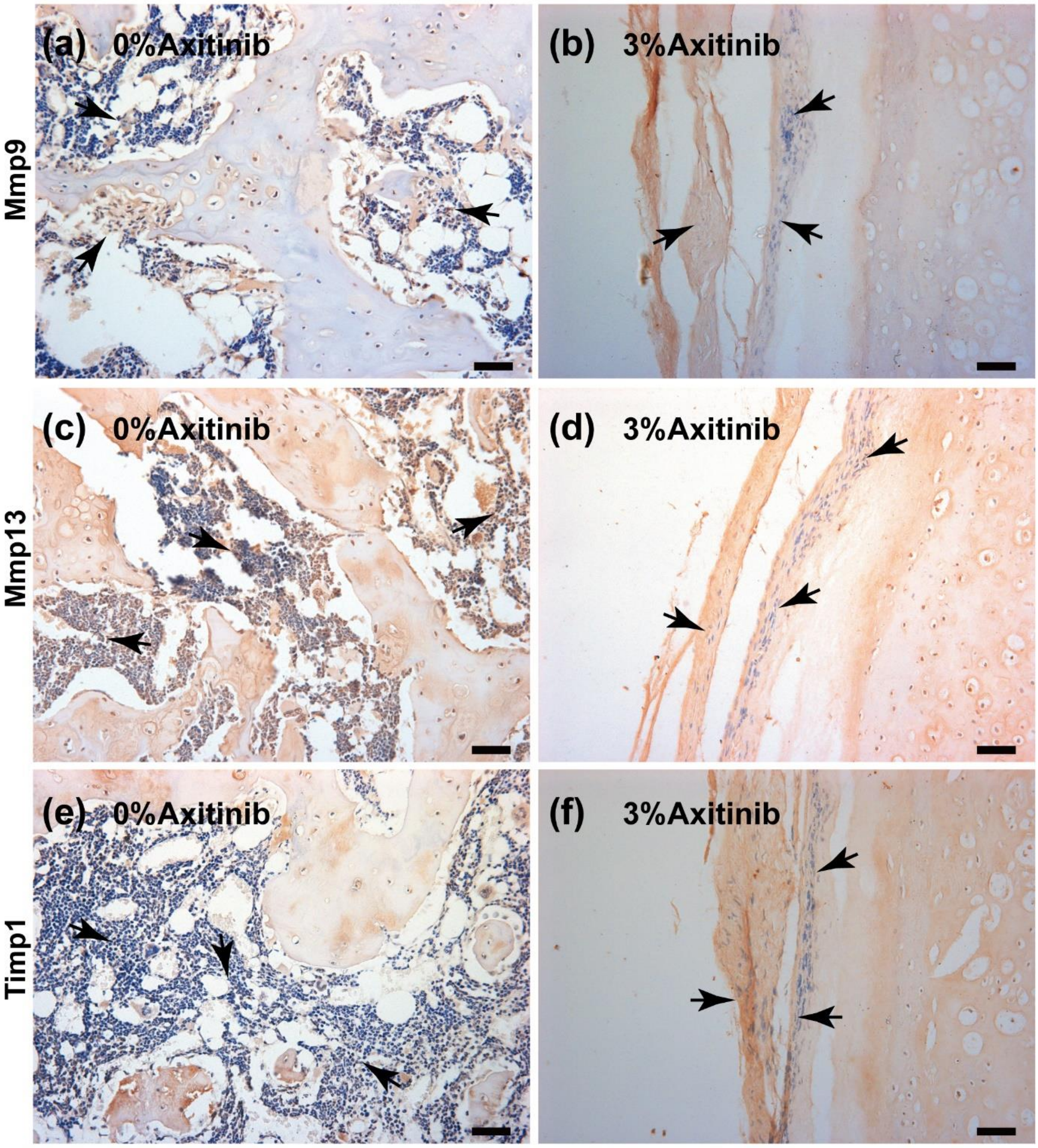

Figure S10. Immunohistochemistry staining of Mmp9, Mmp13, and Timp1 in 0%-Axitinib and 3%-Axitinib groups at 12 weeks post-implantation. Significant positive staining of Mmp9 or Mmp13 was observed in both 0%-Axitinib and 3%-Axitinib groups (a-d). However, the expression of Timp1 in the 0%-Axitinib group (e) was much lower than that in the 3%-Axitinib group (f). Black arrows represented murine tissues surrounding engineered cartilage. Scale bar = 50 μm.

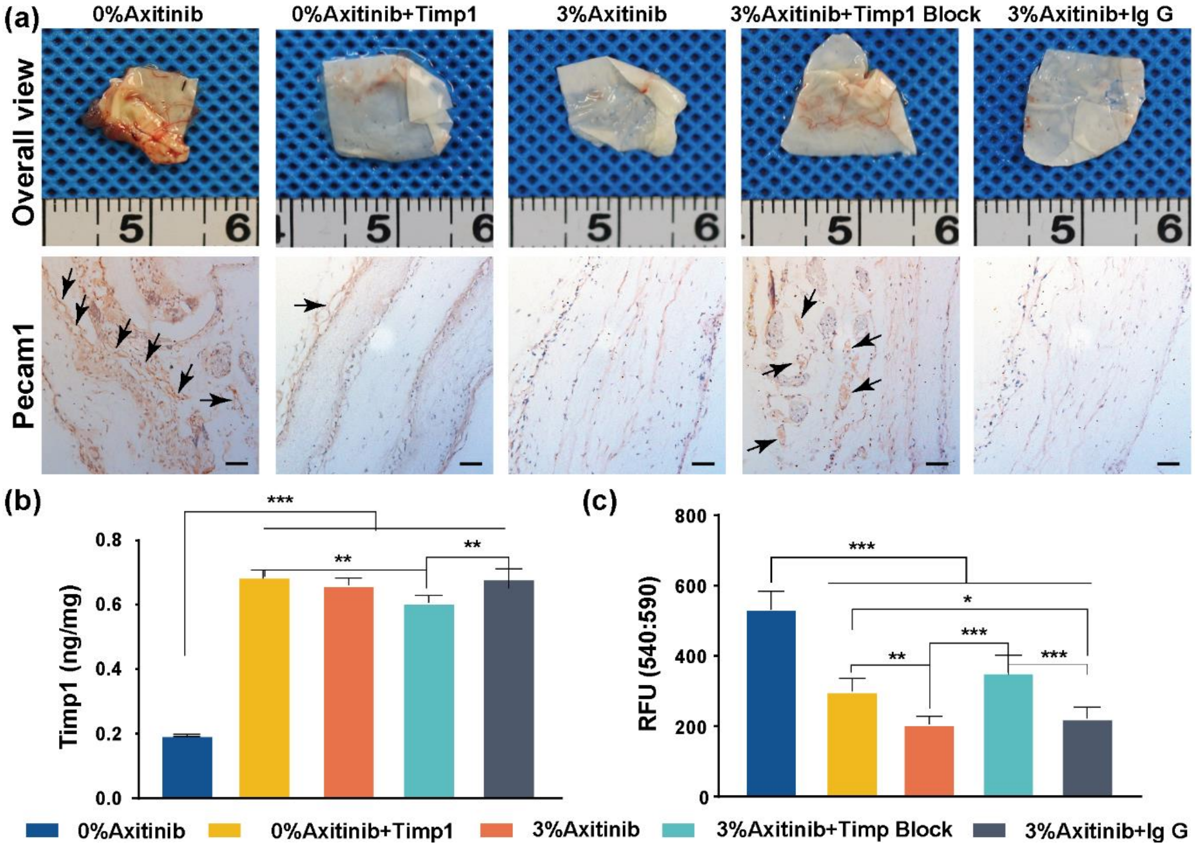

Figure S11. In-vivo modulation of Timp1. (a) Overall view and immunohistochemistry staining of Pecam1 (CD31) of membranes in different groups after two weeks of implantation. Black arrows represented blood vessels. Scale bar = 50 μm. (b) Quantification of murine Timp1 surrounding the membrane. Values represent mean ± SD, n = 4, **P< 0.01, ***P < 0.001. (c) MMP activity of murine tissues surrounding the membrane. Values represent mean ± SD, n = 4, *P < 0.05, **P< 0.01, ***P < 0.001.

Table S1. The general statistics of the in vivo fate of BEC in each group.

Vivo time/grouping	Control	0%-Axitinib	1%-Axitinib	3%-Axitinib	6%-Axitinib
In vivo 4 weeks					
Chondrogenesis case [a)]	4	6	6	6	6
Ossification case [b)]	6	5	1	0	0
Typical bone trabecula [c)]	6	0	0	0	0
In vivo 12 weeks					
Chondrogenesis case	0	3	4	6	6
Ossification case	6	6	2	0	0

Typical bone trabecula	6	5	1	0	0
In vivo 24 weeks					
Chondrogenesis case	0	0	3	6	6
Ossification case	6	6	6	0	0
Typical bone trabecula	6	6	2	0	0

At the scheduled time, 6 samples in each group were evaluated by pathological staining. [a] indicated that typical positive staining of safranin O was observed; [b] indicated that typical positive staining of fast green was observed; [c] indicated that typical bony structures consisting of bone trabecula were observed.

Table S2. Rabbits' primers used for qPCR analysis.

Genes	Forward primer sequences (5'-3')	Reverse primer sequences (5'-3')
Actb	ATCAGCAAGCAGGAGTAT	CAATCTCGTCTCGTTTCTG
Sox9	AGGAGAACACGTTCCCCAAGG	ACCAGCGTCCAGTCGTAGCC
Col2a1	CTCAAGTCCCTCAACAACCAG	TCTATCCAGTAGTCACCGCTCT
Aggrecan	GGTGGTGGTGAAAGGTGTTG	GGTGGAAGCCATCCTCGTAG
Chm-I	ATGACCGAGAACTCGGACAAAGTTC	GAACTTTGTCCGAGTTCTCGGTCAT
Runx2	ATGGCGGGTAATGATGAAAAT	CTCAGATCGTTGAACCTTGCT
Vegfa	GGAGACAATAAACCCCACGAA	GCACGCAGGAAGGCTTGAATA

Table S3. Murine primers used for qPCR analysis.

Genes	Forward primer sequences (5'-3')	Reverse primer sequences (5'-3')
Actb	CCTCACTGTCCACCTTCC	GGGTGTAAAACGCAGCTC
Pecam1	GCCGGAAGACAGAACTAGC	GAGTCCCAGAGCCAGGA
Cdh5	TCACCAGAAAGATCCCAGA	TGGACAGCAAGAGAAAAGG
Timp1	TCACTGTTTGTGGACGGA	AGGCTTCAGGTCATCGG
Mmp9	CACCGGCTAAACCACCTC	CGCCCGACACACAGTAAG
Mmp13	GGACCTTCTGGTCTTCTGG	GCCTTTGGAACTGCTTGT
